# Repetitive Transcranial Magnetic Stimulation Modulates Frontal and Temporal Time-Varying EEG Network in Generalized Anxiety Disorder: A Pilot Study

**DOI:** 10.3389/fpsyt.2021.779201

**Published:** 2022-01-14

**Authors:** Penghui Song, Han Tong, Luyan Zhang, Hua Lin, Ningning Hu, Xin Zhao, Wensi Hao, Peng Xu, Yuping Wang

**Affiliations:** ^1^Department of Neurology, Xuanwu Hospital, Capital Medical University, Beijing, China; ^2^Central Laboratory, Xuanwu Hospital, Capital Medical University, Beijing, China; ^3^Beijing Geriatric Medical Research Center, Beijing, China; ^4^Beijing Key Laboratory of Neuromodulation, Beijing, China; ^5^Neuroscience Graduate Program, University of Cincinnati College of Medicine, Cincinnati, OH, United States; ^6^Division of Behavioral Medicine and Clinical Psychology, Cincinnati Children's Hospital Medical Center, Cincinnati, OH, United States; ^7^Center for Information in Medicine, University of Electronic Science and Technology of China, Chengdu, China; ^8^Beijing Institute for Brain Disorders, Collaborative Innovation Center for Brain Disorders, Capital Medical University, Beijing, China

**Keywords:** generalized anxiety disorder, rTMS, DLPFC, TMS-EEG, time-varying EEG networks

## Abstract

Generalized Anxiety Disorder (GAD) is a highly prevalent yet poorly understood chronic mental disorder. Previous studies have associated GAD with excessive activation of the right dorsolateral prefrontal cortex (DLPFC). This study aimed to investigate the effect of low-frequency repetitive transcranial magnetic stimulation (repetitive TMS, rTMS) targeting the right DLPFC on clinical symptoms and TMS-evoked time-varying brain network connectivity in patients with GAD. Eleven patients with GAD received 1 Hz rTMS treatment targeting the right DLPFC for 10 days. The severity of the clinical symptoms was evaluated using the Hamilton Anxiety Scale (HAMA) and the Hamilton Depression Scale (HAMD) at baseline, right after treatment, and at the one-month follow-up. Co-registration of single-pulse TMS (targeting the right DLPFC) and electroencephalography (TMS-EEG) was performed pre- and post-treatment in these patients and 11 healthy controls. Time-varying brain network connectivity was analyzed using the adaptive directed transfer function. The scores of HAMA and HAMD significantly decreased after low-frequency rTMS treatment, and these improvements in ratings remained at the one-month follow-up. Analyses of the time-varying EEG network in the healthy controls showed a continuous weakened connection information outflow in the left frontal and mid-temporal regions. Compared with the healthy controls, the patients with GAD showed weakened connection information outflow in the left frontal pole and the posterior temporal pole at baseline. After 10-day rTMS treatment, the network patterns showed weakened connection information outflow in the left frontal and temporal regions. The time-varying EEG network changes induced by TMS perturbation targeting right DLPFC in patients with GAD were characterized by insufficient information outflow in the left frontal and temporal regions. Low-frequency rTMS targeting the right DLPFC reversed these abnormalities and improved the clinical symptoms of GAD.

## Introduction

Generalized anxiety disorder (GAD) is a common and debilitating mental disorder; its prevalence rate was found to be 5.7% in an epidemiological survey (1, 2). Patients with GAD mainly present with difficulty in mood regulation and have unrealistic, excessive, and uncontrollable worries about daily affairs (for no reason and for at least 6 months) GAD may be accompanied by dysfunction, such as fatigue, difficulty concentrating, irritability, muscle tension, sleep disturbances, etc (3, 4). The treatment of GAD is still based on drug therapy, supplemented by psychological counseling (5–7); however, the side effects, for instance, delayed movement or dizziness, often lead to treatment cessation (8). Despite the many drug options available, almost 40% of patients with GAD do not respond to pharmacologic treatment (9). Therefore, new treatments for GAD are urgently needed.

Transcranial magnetic stimulation (TMS) is a non-invasive, effective brain stimulation technique that can activate cortical neurons; and its principle is based on Faraday's electromagnetic induction theory (10, 11). Repetitive TMS (rTMS) can reduce or increase cortical excitability depending on the stimulation frequency (12): low-frequency rTMS (≤1HZ) can reduce the excitability of the motor cortex while high-frequency rTMS (≥5HZ) can excite the adjacent cerebral cortex (13). rTMS has been successfully applied in the treatment of anxiety, depression, epilepsy, stroke, and other neurological and psychotic disorders (14–20).

Co-registration of TMS and electroencephalography (TMS-EEG) is a multimodal imaging technique for the direct and non-invasive exploration of cortical reactivity (21, 22). The technique can assess a variety of neurophysiological processes, including cortical responses, local excitation and inhibition, oscillatory activity, effective connectivity, and neuroplasticity, as well as provide important information about the transmission of activity throughout the brain (23). One of the main advantages of TMS-EEG is that it can be used to simultaneously assess the different neurophysiological characteristics of the cortical areas through a time-varying EEG network (24–26).

Recent evidence from neuroimaging studies strongly suggests that mood regulation in patients with GAD is associated with abnormalities in neural circuits of the frontal limbic region, including the dorsolateral prefrontal cortex (DLPFC) (27). The DLPFC plays a central role in emotional regulation by connecting the cortical and subcortical regions (for example, the dorsal anterior cingulate cortex, inferior frontal gyrus, ventral anterior cingulate cortex, and ventral anterior cingulate cortex) (28). Patients with GAD showed greater connectivity between the limbic and prefrontal regions than healthy controls (29).

This study aimed to investigate the effect of low-frequency rTMS targeting the right DLPFC on clinical symptoms and TMS-evoked time-varying brain network connectivity in patients with GAD.

## Subjects and Methods

### Subjects

Eleven patients with GAD (6 men, mean age = 42.1 ± 9.0 years) were recruited between July 2015 and January 2016 from the Department of Neurology, Xuanwu Hospital, Capital Medical University. The inclusion criteria for the patients with GAD were as follows: (1) meeting the diagnostic criteria for generalized anxiety disorder in the DSM-V; (2) aged between 18 and 55 years; (3) having a Hamilton anxiety scale (HAMA) score >14; (4) patients who had taken anti-anxiety drugs did not need to stop taking them, but the medication frequency and dose needed to remain unchanged in the 1 month preceding the experiment; (5) having no abnormalities on physical examination of the nervous system; (6) being right-handed. The exclusion criteria for the GAD group were as follows: (1) having other types of anxiety diagnosed based on the DSM-V; (2) scoring >20 on the Hamilton depression scale (HAMD); (3) having secondary anxiety due to other organic diseases; (4) having a history of brain surgery and epilepsy; (5) having metallic foreign bodies, such as cardiac pacemakers and stents; (6) being a pregnant or lactating patient. Furthermore, 11 healthy subjects (6 men, mean age = 34.5 ± 9.6 years) matched with the GAD group in terms of gender and age were recruited into the control group. This study was approved by the Ethics Committee of Xuanwu Hospital, Capital Medical University. All subjects provided informed consent to participate in this study.

### Neuropsychological Assessment

Each patient with GAD was assessed before and right after rTMS treatment and at the follow-up visit (1 month) using the Hamilton Anxiety Scale (HAM-A) (30) and the Hamilton Depression Rating Scale (HAM-D, 17 items) (31).

### Measurement of the Resting Motor Threshold

Single-pulse TMS was applied with a figure-of-eight coil (70 mm diameter) connected to a monophasic Magstim stimulator (Magstim Company Ltd., London, UK) to measure the resting motor threshold (rMT), which was defined as the lowest stimulation intensity that could produce at least five motor-evoked potentials with wave amplitudes >50 μV among 10 trials in the right first dorsal interosseous muscle. The surface electromyography was recorded using disc-shaped Ag-Cl electrodes that were placed in a tendon-belly arrangement. The stimulating coil was positioned tangentially to the skull with the coil handle pointing backward and laterally at 45° from the anteroposterior axis.

### TMS-EEG Data Acquisition

Twenty-minute TMS-EEG data were acquired using a magnetic field-compatible EEG amplifier (Yunshen Ltd, Beijing, China) digitized with a sampling rate of 1,024 Hz and an electrode cap with 32 TMS-compatible electrodes positioned according to the 10–20 system (Greentek Ltd, Wuhan, China). The electrode impedances were maintained below 5 kΩ. The AFz was used as the grounding electrode, and the nasal tip electrode served as the reference. One hundred and twenty single-pulse TMS stimuli were applied to the right DLPFC (corresponding to F4 points on the subject's scalp, according to the international 10–20 system) at 90% RMT. Each stimulus was applied at an interval of 4 s. The subjects were asked to stay still and have their eyes closed throughout data acquisition. They were also provided with earplugs to block out ambient and coil discharge noises.

### rTMS Treatment

rTMS treatment was administered to all patients with GAD using Magstim Rapid 2 stimulator (Magstim Company Ltd., London, UK). The stimulation site was the right DLPFC (corresponding to F4 points on the subject's scalp, according to the international 10–20 system). The coil plane was tangential and was kept parallel to the scalp, with the coil handle facing the occipital side. The following stimulus parameters were used: frequency, 1 Hz; intensity, 90% RMT; number of stimuli, 1,500 per day for 10 consecutive days.

### TMS-EEG Data Preprocessing

MATLAB (R2015b, The Mathworks, USA) was used for EEG data preprocessing and time-varying network analysis. First, EEG data was imported and the filtering bandwidth was adjusted to 3–30 Hz, with the data sampling rate reduced by 8 times to 128 Hz. EEG data from 1,000 ms before to 2,000 ms after each stimulus point were intercepted as data segments, and about 80–100 data segments were retained for each subject's EEG data.

### Time-Varying EEG Network Analysis

#### Adaptive Directed Transfer Function and the Multivariable Adaptive Autoregressive Model

The adaptive directed transfer function (ADTF) was based on the multivariable adaptive autoregressive model (base on the preprocessed TMS-EEG data) (32):


(1) $$\begin{array}{r} X\left(t\right)=\overset{P}{\underset{i=1}{\sum }}\Lambda \left(i,t\right)X\left(t-i\right)+E\left(t\right) \end{array}$$

Type: *X*(*t*) was the vector data that varied over time, Λ (*i, t*) was the time-varying model coefficient matrix that can be determined by the Kalman filter method (33), *E*(*t*) was multivariate independent white noise, and *P* was the optimal order of the model that can be determined by the Schwarz Bayesian criterion. We then took the Fourier transform of (1):


(2) $$\begin{array}{r} \begin{array}{c} \Lambda \left(f\right)X\left(f\right)=E\left(f\right)\\ \text{X}\left(f\right)={\;\Lambda \;}^{-1}\left(f\right)E\left(f\right)=H\left(f\right)E\left(f\right) \end{array} \end{array}$$

In the formula: $\Lambda \left(f\right)=\sum_{k=0}^{p}\Lambda_{k}e^{-j2\pi f\Delta tk}\left(\Lambda_{k=0}=I\right)$, *H*(*f*) was the transfer coefficient matrix, corresponding to the time-varying model coefficient matrix Λ(*i, t*), and we could get the time-varying transfer coefficient matrix *H*(*f*, *t*). The element *H*_*ij*_(*f*, *t*) represented the connection relationship between the element *J* and the element *I* at frequency *f* and time *t*. The ADTF value could be expressed as follows:


(3) $$\begin{array}{r} ADTF_{ij}\left(f,t\right)={\left|H_{ij}\left(f,t\right)\right|}^{2} \end{array}$$

Standardized as:


(4) $$\begin{array}{r} \;\gamma {\;}_{ij}^{2}\left(f,t\right)=-\frac{{\left|H_{ij}\left(f,t\right)\right|}^{2}}{\overset{n}{\underset{m=1}{\sum }}{\left|H_{im}\left(f,t\right)\right|}^{2}} \end{array}$$

The above equation described the directional causal relationship between elements *J* and *I* at time *T*; it was an effective relationship. In order to calculate all the information flowing from one node to another in a particular frequency band, it was usually possible to combine all $\gamma_{ij}^{2}$ in that frequency band:


(5) $$\begin{array}{r} \;\Theta {\;}_{ij}^{2}\left(t\right)=\frac{\overset{f-2}{\underset{k=f_{1}}{\sum }}\;\gamma {\;}_{ij}^{2}\left(k,t\right)}{f_{2}-f_{1}},\;\Theta {\;}_{ij}^{2}\in \left[0,1\right] \end{array}$$

#### Time-Varying EEG Network Patterns

MATLAB (R2015b) was utilized to identify the dynamic EEG network patterns in healthy subjects and pre- or post- the rTMS treatment.

(1) Calculate 40 time-varying adtf matrices from 360ms before the stimulation point to 40ms before the stimulation point of each data segment in the 3–30 Hz, average all data segments to obtain the baseline time-varying ADTF matrix of each subject, and then average 40 time points in this period to obtain the average baseline ADTF matrix of each subject.

(2) Calculate 246 time-varying ADTF matrices from 60 to 2,000 ms after the stimulation point of each data segment in the 3–30 Hz, and average all data segments to obtain the time-varying ADTF matrix of each subject.

(3) Subtract the average baseline ADTF matrix from the time-varying ADTF matrix of each subject to obtain the time-varying ADTF matrix after baseline correction.

(4) Take the baseline corrected ADTF value of the first 10% of the minimum negative value at each sampling time point of 60–2,000ms, and draw the time-varying brain network diagram of weakened connection used Brain_graphic (24).

### Statistical Analysis

Demographic and clinical variables were compared using between-group two-sample, two-tailed *t*-tests or chi-squares. The HAMA and HAMD scores of GAD patients at three time points (before treatment, at the end of treatment, and 1 month after the end of treatment) were analyzed using repeated-measures ANOVA. The effects were considered significant if *p* < 0.05.

## Results

### Neuropsychological Characteristics

We enrolled a total of 22 subjects into the study: 11 patients with GAD (6 men, mean age = 42.1 ± 9.0 years) and 11 healthy subjects (6 men, mean age = 34.5 ± 9.6 years). The healthy subjects were matched with the patients with GAD in terms of gender and age. All the subjects completed the entire study without adverse events. The HAMA and HAMD scores significantly decreased after rTMS treatment and 1 month after treatment (Table 1).

**Table 1:** Neuropsychological characteristics of GAD.

**Variables**	**Before treatment**	**After treatment**	**1 month after treatment**
HAMA	21.45 ± 4.13	11.27 ± 4.36^*^	11.36 ± 3.72^#^
HAMD	13.45 ± 4.66	8.73 ± 3.72^*^	8.27 ± 3.29^#^

*Data are presented as mean ± SD*.

* *P < 0.05 vs. before treatment in the same group*,

# *P < 0.05 vs. before treatment in the same group. GAD, Generalized anxiety disorder; HAMA, Hamilton Anxiety Scale; HAMD, Hamilton Depression Scale*.

### Time-Varying EEG Network Patterns

**Figure 1:**
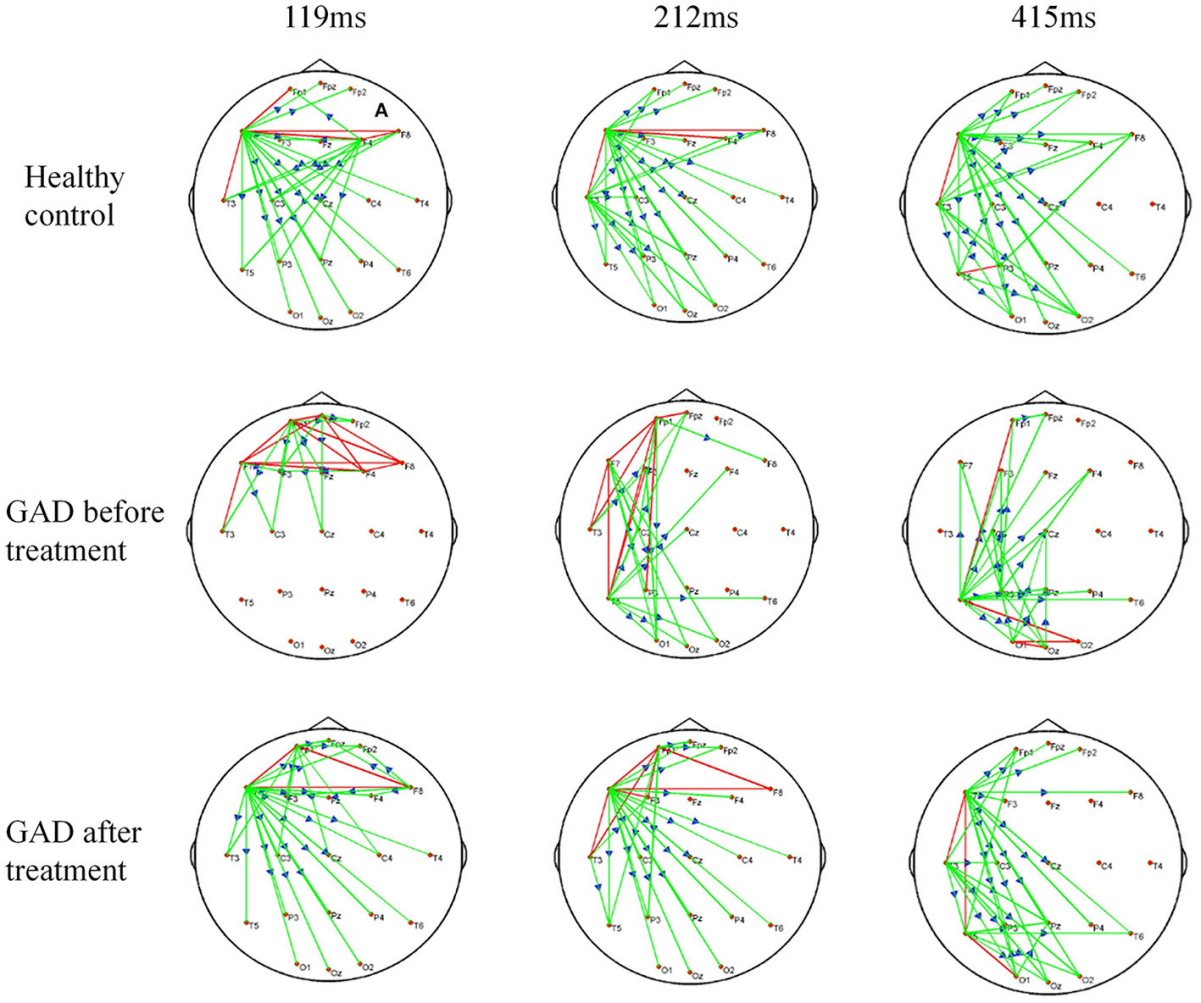
The time-varying EEG network connections after single-pulse TMS stimulation compared to before stimulation. Time, after single TMS. Green lines, decreased infor mation; blue arrows, the direction of information flow; Red lines, two-way decreased information; GAD, Generalized anxiety disorder.

From the above time-varying network analysis, Figure 1 shows the EEG network patterns of the patients with GAD before and after treatment as well as those of the healthy controls. In the healthy controls, the time-varying EEG network after single-pulse TMS of the right DLPFC showed the hub node on the left frontal (119 and 212 ms) and left mid-temporal (212 ms and 415 ms) weakened connection patterns. Compared with that of the healthy controls, the time-varying EEG network after single-pulse TMS targeting the right DLPFC of the patients with GAD before therapy showed the hub node on the left frontal pole (119 ms) and left posterior temporal pole (212 and 415 ms) weakened connection patterns. Compared with the patterns before treatment, the time-varying EEG network after single-pulse TMS targeting the right DLPFC of the patients with GAD after rTMS treatment showed the hub node on the left frontal (119 and 212 ms) and left temporal (212 and 415 ms) weakened connection patterns. The results show that the time-varying network pattern after treatment is very similar to the healthy subject, indicating that rTMS treatment promotes the restoration of brain network connections.

## Discussion

### Summary of Key Findings

To the best of our knowledge, this study was the first to use time-varying EEG networks to investigate the underlying neural connection mechanisms of GAD. In the present study, we found that low-frequency rTMS stimulating the right DLPFC was effective at improving the symptoms of GAD, and the abnormal time-varying EEG networks in GAD showed a trend toward normalization after rTMS treatment. Furthermore, this effect was sustained: at 1 month follow-up visit, GAD continued to report fewer anxiety symptoms as the HAMA and HAMD scores continued to significantly decline from those reported at the end of treatment. This study demonstrates that rTMS does have potential as an effective augmentative treatment in GAD.

### Comparison With Previous Studies

The prefrontal lobe is an important part of the neural loop of emotional processing. It plays an important regulatory role through its round-trip connection with the temporal lobe and limbic system, in which the DLPFC is the pivotal brain region. Using fMRI, Bystritsky et al. found that the right DLPFC of patients with GAD was abnormally activated, and they recruited 10 patients with GAD to undergo low-frequency rTMS therapy for a total of 6 times for 3 weeks (1 Hz, 90% rMT, 900 stimuli per time), with the stimulation targeting the right DLPFC; the HAMA score significantly reduced at the end of the treatment (34). Gretchen et al. (35) stimulated the right DLPFC with rTMS (1 Hz, 90% RMT) and observed that after active rTMS treatment, the activity of the right DLPFC significantly improved and there were improvements in self-reported emotion regulation difficulties at posttreatment and at the 3-month follow-up in the active group only. In the present study, patients with GAD were treated with low-frequency rTMS (1 Hz, 90% RMT), targeting the right DLPFC for 10 days. The results showed that the clinical symptoms of 11 patients with GAD improved after treatment, and the scores of HAMA and HAMD significantly decreased compared to those before treatment. The curative effect lasted at least 1 month.

### Interpretation of Findings

The left and right hemispheres of the human brain are functionally asymmetrical. According to the theory of the titer model of brain emotions, the right hemisphere plays a leading role in the processing of negative emotions, while the left hemisphere is mainly responsible for processing positive emotions (36). A study using fMRI found that showing subjects pictures with obvious emotional colors led to increased blood flow in the bilateral anterior cingulate gyri, DLPFC, amygdala, and anterior temporal brain (37). Negative images more significantly activated the relevant brain regions of the right hemisphere, and positive meanings significantly activated the relevant brain regions in the left hemisphere (38). Although our study has shown that rTMS can improve GAD symptoms through the regulation of the DLPFC, the exact neurobiological mechanisms remain unclear. We consider that GAD is a disease of abnormal brain function in which the different cortex areas have abnormal connections.

Abnormal functional connectivity of GAD has been reported widely in fMRI studies. Compared with healthy controls, the function connectivity of the right medial prefrontal gyrus of the default mode network and the superior temporal gyrus of the salience network increased significantly in the GAD patients (39). We used the adaptive directed transfer function to analyze TMS-EEG signals, as well as to prove the existence of network abnormality in GAD. Compared with the healthy controls in patients with GAD, the time-varying EEG network showed that the right DLPFC has insufficient inhibition of information outflow from the left frontal and temporal regions, and that the abnormal activation of the left temporal lobe leads to overreaction to external stimulus processing. Therefore, low-frequency rTMS is administered to the right DLPFC to inhibit its activity and promote the restoration of the information outflow trend toward the normal.

Excessive and uncontrollable worry is the core symptom of GAD, the pathological worry pattern might be linked with alterations of fronto-limbic regions, such as the DLPFC and amygdala, to handle the external threat through the heightened arousal and distress state (40). RTMS strengthened the GAD processing advantage of positive emotions by activating information outflow from the left frontal lobe. As a result, the symptoms of anxiety and depression significantly improved. This observation may support the hypothesis that GAD may be a disorder of brain functional connectivity, and rTMS treatment could reverse this abnormity.

### Strengths and Limitations

This study had several limitations. First, the sample size was relatively small; a larger sample size is needed in further investigations. Second, we should add a sham stimulation group as a control. Third, further studies need to use the neuro-navigated system to locate DLPFC to improve the accuracy of stimulation. Last but not least, we only used HAMA to measure anxiety in patients with GAD; in the subsequent studies, we will add the State-Trait Anxiety Inventory (STAI), a more sensitive inventory in case of GAD because it measures both state and trait anxiety.

## Conclusion

The present study was designed to determine the effectiveness of low-frequency rTMS treatment for GAD. We found that the curative effect lasted at least 1 month. Our study revealed that the right DLPFC in GAD has insufficient inhibition of information outflow in the left frontal and temporal regions. Low-frequency rTMS treatment targeting the right DLPFC may reverse these abnormal changes and improve the symptoms of anxiety.

## Data Availability Statement

The raw data supporting the conclusions of this article will be made available by the authors, without undue reservation.

## Ethics Statement

The studies involving human participants were reviewed and approved by Ethics Committee of Xuanwu Hospital, Capital Medical University. The patients/participants provided their written informed consent to participate in this study.

## Author Contributions

PX and YW contributed to the conception of the study. HT, NH, and XZ performed the experiments. LZ and HT performed the data analyses. PS and WH wrote the manuscript. HT, HL, and YW helped to perform the analysis with constructive discussions. All authors contributed to the article and approved the submitted version.

## Funding

The study was supported by National Key R&D Program of China (Grant Numbers: 2021YFC2501404, 2019YFC0121200, 2019YFC0121202, 2019YFC0121203, 2018YFC1314500, 2018YFA0108503, and 2016YFC1306302), National Natural Science Foundation of China (Grant Numbers: 82001388, 81771398, and 81801285), and Beijing Municipal Administration of Hospital Clinical Medicine Development of Special Funding Support (Grant Number: ZYLX201706).

## Conflict of Interest

The authors declare that the research was conducted in the absence of any commercial or financial relationships that could be construed as a potential conflict of interest.

## Publisher's Note

All claims expressed in this article are solely those of the authors and do not necessarily represent those of their affiliated organizations, or those of the publisher, the editors and the reviewers. Any product that may be evaluated in this article, or claim that may be made by its manufacturer, is not guaranteed or endorsed by the publisher.

## References

[1] Kessler RC Berglund P Demler O Jin R Merikangas KR Walters EE. Lifetime prevalence and age-of-onset distributions of DSM-IV disorders in the National Comorbidity Survey Replication. Arch Gen Psychiatry. (2005) 62:593–602. 10.1001/archpsyc.62.6.593 15939837

[2] Ströhle A Gensichen J Domschke K. The diagnosis and treatment of anxiety disorders. Dtsch Arztebl Int. (2018) 155:611–20. 10.3238/arztebl.2018.0611 30282583 PMC6206399

[3] DeMartini J Patel G Fancher TL. Generalized anxiety disorder. Ann Intern Med. (2019) 170:ITC49–64. 10.7326/AITC201904020 30934083

[4] Kampman O Viikki M Leinonen E. Anxiety disorders and temperament-an update review. Curr Psychiatry Rep. (2017) 19:27. 10.1007/s11920-017-0779-5 28417269

[5] Bandelow B Zohar J Hollander E Kasper S Möller HJ Zohar J. World Federation of Societies of Biological Psychiatry (WFSBP) guidelines for the pharmacological treatment of anxiety, obsessive-compulsive and post-traumatic stress disorders—first revision. World J Biol Psychiatry. (2008) 9:248–312. 10.1080/15622970802465807 18949648

[6] Daitch C. Cognitive behavioral therapy, mindfulness, and hypnosis as treatment methods for generalized anxiety disorder. Am J Clin Hypn. (2018) 61:57–69. 10.1080/00029157.2018.1458594 29771217

[7] Slee A Nazareth I Bondaronek P Liu Y Cheng Z Freemantle N. Pharmacological treatments for generalised anxiety disorder: a systematic review and network meta-analysis. Lancet. (2019) 393:768–77. 10.1016/S0140-6736(18)31793-8 30712879

[8] Buoli M Caldiroli A Caletti E Paoli RA Altamura AC. New approaches to the pharmacological management of generalized anxiety disorder. Expert Opin Pharmacother. (2013) 14:175–84. 10.1517/14656566.2013.759559 23282069

[9] Baldwin DS Waldman S Allgulander C. Evidence-based pharmacological treatment of generalized anxiety disorder. Int J Neuropsychopharmacol. (2011) 14:697–710. 10.1017/S1461145710001434 21211105

[10] Cirillo P Gold AK Nardi AE Ornelas AC Nierenberg AA Camprodon J. (2019). Transcranial magnetic stimulation in anxiety and trauma-related disorders: a systematic review and meta-analysis. Brain Behav. 9, e01284. 10.1002/brb3.1284 31066227 PMC6576151

[11] Taylor R Galvez V Loo C. Transcranial magnetic stimulation (TMS) safety: a practical guide for psychiatrists. Australas Psychiatry. (2018) 26:189–92. 10.1177/1039856217748249 29338288

[12] Pernia AM Zorzo C Prieto MJ Martinez JA Higarza SG Mendez M. Equipment for repetitive transcranial magnetic stimulation. IEEE Trans Biomed Circuits Syst. (2020) 14:525–34. 10.1109/TBCAS.2020.2981012 32175874

[13] Tracy DK de Sousa DAM Nalesnik N Mao L Lage C Shergill SS. Neuroimaging effects of 1 Hz right temporoparietal rTMS on normal auditory processing: implications for clinical hallucination treatment paradigms. J Clin Neurophysiol. (2014) 31:541–6. 10.1097/WNP.0000000000000098 25462140

[14] Choi KM Jang K Jang KI Um YH Kim M Kim D. The effects of 3 weeks of rTMS treatment on P200 amplitude in patients with depression. Neurosci Lett. (2014) 577:22–7. 10.1016/j.neulet.2014.06.003 24928222

[15] Choi KM Choi S Lee SM Jang K Chae J. Three weeks of rTMS treatment maintains clinical improvement but not electrophysiological changes in patients with depression: a 6-week follow-up pilot study. Front Psychiatry. (2019) 10:351. 10.3389/fpsyt.2019.00351 31231248 PMC6566016

[16] Dionísio A Duarte IC Patrício M Castelo-Branco M. The use of repetitive transcranial magnetic stimulation for stroke rehabilitation: a systematic review. J Stroke Cerebrovasc Dis. (2018) 27:1–31. 10.1016/j.jstrokecerebrovasdis.2017.09.008 29111342

[17] Kiebs M Hurlemann R Mutz J. Repetitive transcranial magnetic stimulation in non-treatment-resistant depression. Br J Psychiatry. (2019) 215:445–6. 10.1192/bjp.2019.75 31014413

[18] Rachid F. Repetitive transcranial magnetic stimulation in the treatment of eating disorders: a review of safety and efficacy. Psychiatry Res. (2018) 269:145–56. 10.1016/j.psychres.2018.08.013 30149272

[19] Tsuboyama M Kaye HL Rotenberg A. Review of transcranial magnetic stimulation in epilepsy. Clin Ther. (2020) 42:1155–68. 10.1016/j.clinthera.2020.05.016 32624320

[20] Yang LL Zhao D Kong LL Sun YQ Wang ZY Gao YY. High-frequency repetitive transcranial magnetic stimulation (rTMS) improves neurocognitive function in bipolar disorder. J Affect Disord. (2019) 246:851–6. 10.1016/j.jad.2018.12.102 30795490

[21] Tremblay S Rogasch NC Premoli I Blumberger DM Casarotto S Chen R. Clinical utility and prospective of TMS-EEG. Clin Neurophysiol. (2019) 130:802–44. 10.1016/j.clinph.2019.01.001 30772238

[22] Kimiskidis K. Transcranial magnetic stimulation (TMS) coupled with electroencephalography (EEG): biomarker of the future. Rev Neurol. (2016) 172:123–6. 10.1016/j.neurol.2015.11.004 26857413

[23] Koch G. The new era of TMS-EEG: Moving towards the clinical practice. Clin Neurophysiol. (2019) 130:791–2. 10.1016/j.clinph.2019.02.004 30827795

[24] Song P Lin H Li S Wang L Liu J Li N. Repetitive transcranial magnetic stimulation (rTMS) modulates time-varying electroencephalography (EEG) network in primary insomnia patients: a TMS-EEG study. Sleep Med. (2019) 56:157–63. 10.1016/j.sleep.2019.01.007 30871961

[25] Song P Lin H Liu C Jiang Y Lin Y Xue Q. Transcranial magnetic stimulation to the middle frontal gyrus during attention modes induced dynamic module reconfiguration in brain networks. Front Neuroinform. (2019) 13:22. 10.3389/fninf.2019.00022 31001103 PMC6456710

[26] Song P Li S Wang S Wei H Lin H Wang Y. Repetitive transcranial magnetic stimulation of the cerebellum improves ataxia and cerebello-fronto plasticity in multiple system atrophy: a randomized, double-blind, sham-controlled and TMS-EEG study. Aging. (2020) 12:20611–22. 10.18632/aging.103946 33085647 PMC7655163

[27] Ochsner KN Silvers JA Buhle JT. Functional imaging studies of emotion regulation: a synthetic review and evolving model of the cognitive control of emotion. Ann N Y Acad Sci. (2012) 1251:E1–24. 10.1111/j.1749-6632.2012.06751.x 23025352 PMC4133790

[28] Diekhof EK Geier K Falkai P Gruber O. Fear is only as deep as the mind allows: a coordinate-based meta-analysis of neuroimaging studies on the regulation of negative affect. Neuroimage. (2011) 58:275–85. 10.1016/j.neuroimage.2011.05.073 21669291

[29] Andreescu C Sheu LK Tudorascu D Gross JJ Walker S Banihashemi L. Emotion reactivity and regulation in late-life generalized anxiety disorder: functional connectivity at baseline and post-treatment. Am J Geriatr Psychiatry. (2015) 23:200–14. 10.1016/j.jagp.2014.05.003 24996397 PMC4234701

[30] Hamilton M. The assessment of anxiety states by rating. Br J Med Psychol. (1959) 32:50–5. 10.1111/j.2044-8341.1959.tb00467.x 13638508

[31] Hamilton M. A rating scale for depression. J Neurol Neurosurg Psychiatry. (1960) 23:56–62. 10.1136/jnnp.23.1.56 14399272 PMC495331

[32] Zhang L Liang Y Li F Sun H Peng W Du P. Time-varying networks of inter-ictal discharging reveal epileptogenic zone. Front Comput Neurosci. (2017) 11. 10.3389/fncom.2017.00077 28867999 PMC5563307

[33] Arnold M Milner XHR Witte H Bauer R Braun C. Adaptive AR modeling of nonstationary time series by means of Kalman filtering. IEEE Transac Biomed Eng. (1998) 45:553–62. 10.1109/10.668741 9581053

[34] Bystritsky A Kaplan JT Feusner JD Kerwin LE Wadekar M Burock M. A preliminary study of fMRI-guided rTMS in the treatment of generalized anxiety disorder. J Clin Psychiatry. (2008) 69:1092–8. 10.4088/JCP.v69n0708 18572984

[35] Diefenbach GJ Bragdon LB Zertuche L Hyatt CJ Hallion LS Tolin DF. Repetitive transcranial magnetic stimulation for generalised anxiety disorder: a pilot randomised, double-blind, sham-controlled trial. Br J Psychiatry. (2016) 209:222–8. 10.1192/bjp.bp.115.168203 27198484

[36] Heller W Nitschke JB Etienne MA Miller GA. Patterns of regional brain activity differentiate types of anxiety. J Abnorm Psychol. (1997) 106:376–85. 10.1037/0021-843X.106.3.376 9241939

[37] Lai CH. Task MRI-based functional brain network of anxiety. Adv Exp Med Biol. (2020) 1191:3–20. 10.1007/978-981-32-9705-0_1 32002919

[38] Alfano KM Cimino CR. Alteration of expected hemispheric asymmetries: valence and arousal effects in neuropsychological models of emotion. Brain Cogn. (2008) 66:213–20. 10.1016/j.bandc.2007.08.002 17928118

[39] Xiong H Guo RJ Shi HW. Altered default mode network and salience network functional connectivity in patients with generalized anxiety disorders: an ICA-based resting-state fMRI study. Evid Based Complement Alternat Med. (2020) 2020:4048916. 10.1155/2020/4048916 32855650 PMC7443230

[40] Meeten F Davey GC Makovac E Watson DR Garfinkel SN Critchley HD. Goal directed worry rules are associated with distinct patterns of amygdala functional connectivity and vagal modulation during perseverative cognition. Front Hum Neurosci. (2016) 10:553. 10.3389/fnhum.2016.00553 27853428 PMC5089972

